# 5-year clinical and radiostereometric analysis (RSA) follow-up of 39 CUT femoral neck total hip prostheses in young osteoarthritis patients

**DOI:** 10.3109/17453674.2012.702392

**Published:** 2012-08-25

**Authors:** Marc J Nieuwenhuijse, Edward R Valstar, Rob G H H Nelissen

**Affiliations:** ^1^Department of Orthopedic Surgery, Leiden University Medical Center, Leiden; ^2^Department of Biomechanical Engineering, Faculty of Mechanical, Maritime, and Materials Engineering, Delft University of Technology, Delft, the Netherlands

## Abstract

**Background:**

As the number of young patients receiving total hip arthroplasty increases, bone-saving implantations facilitating possible future revision, such as the CUT femoral neck prosthesis, are gaining importance. There have been few medium-term results reported for this prosthesis, however, and its migration pattern has not been analyzed.

**Patients and methods:**

39 consecutive CUT femoral neck prostheses were implanted in 32 patients, mean age 37 (17–58) years, with symptomatic osteoarthritis and either less than 55 years of age or with an anatomic anomaly preventing implantation of a diaphyseal stem (n = 1). Patients were followed prospectively using routine clinical examination and radiostereometric analysis (RSA) at 6, 12, 26, and 52 weeks postoperatively and annually thereafter. This study evaluated the 5-year follow-up results.

**Results:**

The mean Harris hip score increased from 26 (3–51) points preoperatively to 84 (66–98), 86 (55–98), and 87 (47–98) points at 3, 12, and 60 months. 3 stems were revised: 1 after luxation following excessive subsidence due to an undersized component and 2 due to persistent strong thigh pain. 5-year survival was 95% (95% CI: 87–100). Initial migration varied widely in magnitude; median total tip migration was 0.42 mm (0.09–9.4) at 6 weeks, 0.92 mm (0.18–5.9) at 1 year, and 1.10 mm (0.13–6.4) at 5 years. Even after high initial migration, stabilization was achieved in 31 of the 35 RSA-evaluable implants. 3 prostheses showed progressive continuous migration throughout the entire follow-up period, and were considered to be loose, suggesting reduced long-term survival.

**Interpretation:**

Currently, we cannot recommend the CUT femoral neck prosthesis as a routine treatment option in (young) patients requiring THA. The CUT prosthesis may not reach the 90% survival benchmark at 10 years, and the prosthesis is difficult to implant. If initial stabilization is achieved, however, aseptic loosening is unlikely. A good clinical outcome was seen in the surviving prostheses. We will continue to follow this patient group.

As both the number of young patients receiving total hip arthroplasty and the current life expectancy are increasing, the frequency of revision surgery is also expected to increase (Walker et al. 2005, Huo et al. 2008). To maximize the potential for successful possible future revision, several prostheses that require only minor bone loss for implantation have been developed (Morrey 1989, Walker et al. 2005, Röhrl et al. 2006). One of these prostheses is the femoral neck prosthesis CUT (ESKA Implants, Lübeck, Germany) (Thomas et al. 1999, 2004).

The CUT prosthesis allows cementless metaphyseal fixation of the femoral component. Only the femoral head is resected, and the femoral neck is retained to support the implant. Mechanical and experimental studies of the CUT prosthesis showed a favorable strain distribution with prevention of the strain decrease and subsequent bone resorption in the proximal femur commonly seen with diaphyseal stems (Koebke et al. 2000, Specht et al. 2003, Decking et al. 2006, 2008).

Only short and medium-term clinical results of the CUT prosthesis have been published (Thomas et al. 2004, Ender et al. 2007, Rudert et al. 2007, Ishaque et al. 2009, Steens et al. 2010). In these reports, the reported medium-term survival varied from 50% to 98%. This was mainly due to varying revision rates for aseptic loosening, which accounted for the majority of failures.

Using radiostereometric analysis (RSA), migration—and therefore fixation—of prostheses can be assessed with high accuracy. Excessive early migration of implants is associated with long-term aseptic loosening, and RSA is a suitable tool for early evaluation of long-term implant performance (Kärrholm et al. 1994, Ryd et al. 1995, Hauptfleisch et al. 2006, Nelissen et al. 2011).

In this paper we report the 5-year clinical and RSA follow-up results of 39 consecutive CUT prostheses implanted for symptomatic osteoarthritis in a young patient population. We evaluated prosthesis survival, estimated the rate of aseptic loosening as determined by RSA, and assessed the influence of implant positioning on migration.

## Patients and methods

Between July 2002 and February 2007, 39 consecutive CUT prostheses were implanted in 32 consecutive patients (12 male, 20 female; 7 bilateral) for symptomatic osteoarthritis who were either less than 55 years of age or had an anatomical anomaly preventing implantation of a regular diaphyseal stem. Mean age was 37 (17–58) years. The preoperative diagnosis in the patients younger than 55 years was primary osteoarthritis in 9 hips and secondary osteoarthritis in 29 hips. Osteoarthritis was secondary to osteonecrosis of the femoral head (9), rheumatoid arthritis (6), juvenile idiopathic arthritis (5), developmental dysplasia (4), morbus Morquio (2), epiphysiolysis (1), septic monoarthritis (1) and monoarthritis of unknown cause (1). In 1 patient, the anatomical appearance necessitated placement of this short-stemmed prosthesis: a 58-year-old woman with osteoarthritis secondary to congenital arthrogryposis multiplex who had a fork-deformed femur.

The CUT prosthesis is made of a CoCrMo alloy. The femoral component consists of a body and a modular conus with various angles and lengths in order to restore leg length and offset. The surface of the prosthesis has a macroporous spongious metal surface structure to facilitate bone ingrowth. 2 experienced orthopedic surgeons (RGHHN and HJO) operated on the patients using a lateral approach in the lateral decubital position. The senior author learned the technique from one of the prosthesis designers. In 37 cases, the CUT prostheses was combined with the standard press-fit cup (ESKA Implants, Lübeck, Germany) and in 2 cases it was combined with a press-fit Mallory Head cup (Biomet, Warsaw, IN). In all cases, a polyethylene liner was combined with a ceramic head.

**Figure 1.**
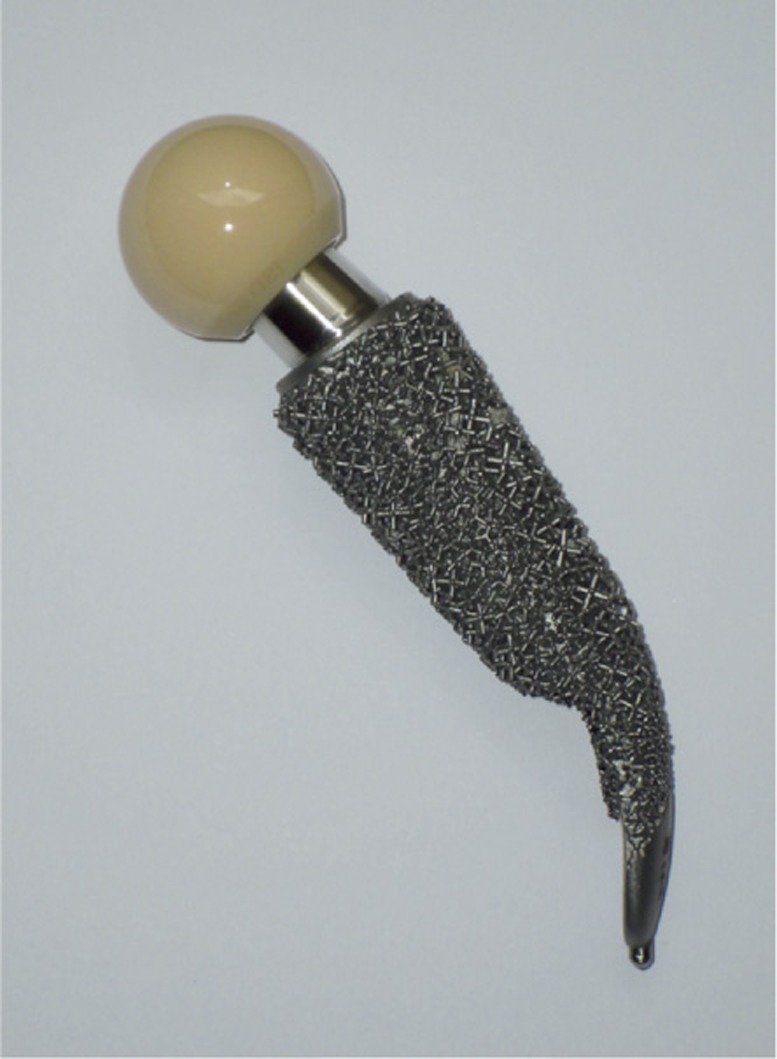
The CUT femoral neck prosthesis. Note the RSA marker at the distal tip of the prosthesis.

For RSA analysis, 6–10 1-mm tantalum balls (Industrial Tectonics, Ann Arbor, MI) were inserted into the proximal femur during surgery. Furthermore, the implant manufacturer attached 1 marker at the distal tip of the hook-shaped end of the prosthesis (Figure 1). Attachment of additional markers to the prosthesis without fundamentally altering the design of the prosthesis was attempted, but proved unsuccessful. Also, usage of the femoral head as an additional prosthesis marker, which is common in RSA of femoral components, was not feasible due overprojection of the metal-backed acetabular component. Thus, only translations of the tip could be determined. The first RSA radiographs were taken in the first postoperative week before ambulation (median 4 (1–7) days postoperatively). The patients were allowed minimal weight bearing using 2 crutches in the first 3 postoperative weeks, partial weight bearing using 1 crutch during the next 3 postoperative weeks, and full weight bearing thereafter.

Patients were evaluated preoperatively and postoperatively at 6 weeks, 3 months, 6 months, 1 year, and annually thereafter. At each evaluation, the Harris hip score and RSA radiographs were obtained. Conventional anteroposterior and lateral radiographs were obtained at 6 weeks, 1 year, 2 years and 5 years of follow-up. None of the patients were lost to follow-up, but 1 patient was unable to attend the most recent follow-up due to pregnancy. Mean length of follow-up was 7.0 (4.8–9.5) years. 37 5years prostheses had at least 5 years of follow-up.

Marker-based RSA measurements were performed (MB-RSA software; Medis Specials, Leiden, the Netherlands) (Kaptein et al. 2003). The first RSA examination served as the reference for all further examinations; all evaluations are related to the position of the prosthesis relative to the bone at that time. Migration is expressed along the longitudinal, transverse, and sagittal axes. Accuracy of individual RSA measurements was given by the limits of the 95% prediction interval of the accuracy of zero motion (Valstar et al. 2005), calculated as 1.96√(∑d^2^/2n) (Ranstam et al. 2000) using 25 double examinations obtained at 1-year follow-up. Individual measurement accuracy was ± 0.08mm for medial-lateral translation, ± 0.08 mm for cranial-caudal translation, ± 0.22 mm for anterior-posterior translation, and ± 0.25 mm for total translation.

In 3 of 39 prostheses, there were insufficient or incorrectly placed RSA markers and these patients were unsuitable for RSA analysis. In addition, 1 patient refused further RSA examinations after the 6-week postoperative RSA radiograph and this patient was excluded from migration analysis. In these 4 patients, routine clinical and radiographic follow-up was performed. Thus, RSA follow-up was complete in 35 of 39 prostheses. For all examinations, the rigid body error was below 0.35 and the condition number was below 56; these values satisfy the marker stability and distribution criteria according to the RSA guidelines of Valstar et al. (2005). No examinations had to be excluded.

**Figure 2.**
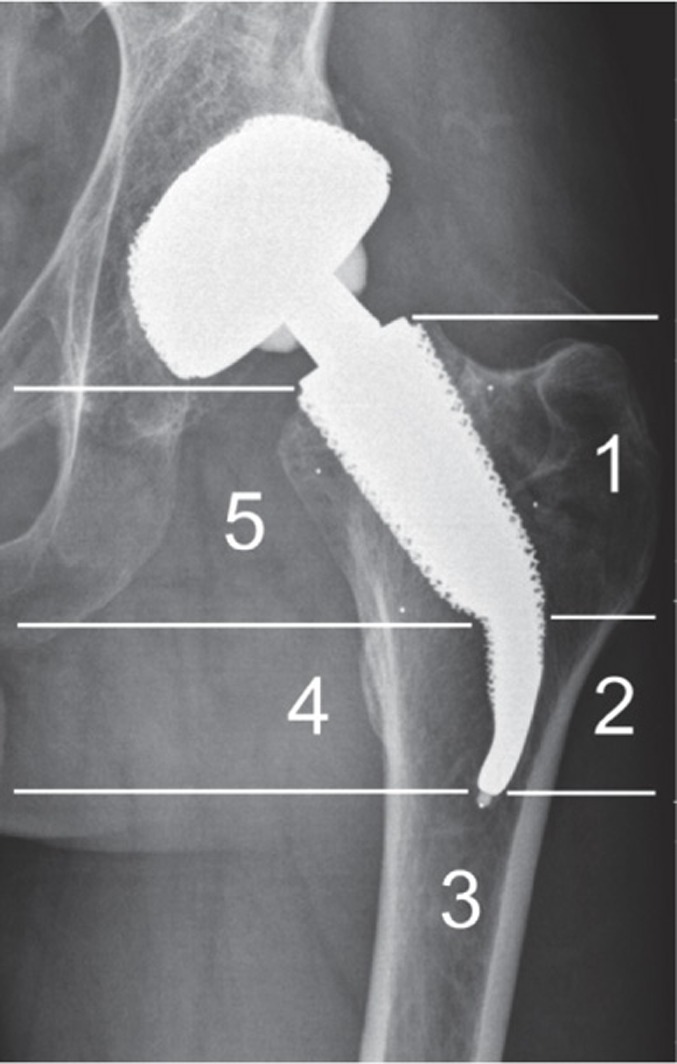
5 assessment zones around the CUT femoral neck prosthesis (modified according to Gruen).

Preoperative offset and caput collum diaphyseal angle (CCD angle) were measured on conventional preoperative pelvic radiographs. On conventional postoperative pelvic radiographs, the amount of femoral neck resection (no femoral neck engagement, less than 50% resection, or more than 50% resection), alignment of the stem, postoperative offset and CCD angle, distance of the proximal stem to the medial femoral cortex, and distance of the distal stem to the lateral femoral cortex were determined. The presence of radiolucent lines and stress shielding was assessed in 5 zones around the stem (Figure 2).

Informed consent from the patients and approval of the institutional review board were obtained for the study.

### Statistics

Values are reported as mean (SD) and range, or as median (range). Estimates are reported as mean with 95% confidence interval (CI).

The Kaplan-Meier method was used to estimate survival of the prostheses. Initial (0–2 years) and steady-state (≥ 2 years) migration were analyzed using a linear mixed model with random slope and random intercept, which accounts for the repeated measurements of migration over time and the correlation of these measurements in patients. Variables in the analysis were: days to the first postoperative RSA examination, alignment of the stem, postoperative CCD angle, change in CCD angle and offset, categorized distance (0–2, 2–4, 4–6, > 6 mm) to the medial and lateral cortex, the amount of femoral neck resection, and the occurrence of stress shielding (steady-state migration only). Any p-value of < 0.05 was considered statistically significant. We used SPSS statistical software version 17.0.

## Results

### Clinical results

The mean Harris hip score increased from 26 (SD 16, range: 3–51) points preoperatively to 84 (SD 8.4, 66–98) points at 3 months, 86 (SD 12, 55–98) points at 1 year, and 88 (SD 14, 47–98) points at 5 years (p < 0.001). 34 of 39 THAs had a good or very good result (with a mean score during 1–5 years of between 81 and 100 points).

The mean preoperative CCD angle was 138° (SD 7.9, 117–154). The mean preoperative offset was 35 (SD 11, 10 – 65) mm. Postoperatively, the mean CCD angle was 138° (SD 7.7, 119–153) and the mean offset was 35 (SD 8.1, 16 – 59) mm. The mean inclination angle of the stem was 151° (SD 8, 136–167). Mean distance from the medial cortex and lateral cortex was 2.8 mm (SD 1.5, 0–6) and 1.8 mm (SD 1.9, 0–8), respectively. The femoral neck was correctly resected in 32 prostheses (no engagement in 12 prostheses, less than 50% engagement in 19 prostheses, and more than 50% engagement in 8 prostheses).

During follow-up, radiolucent lines < 1 mm were noted in zone 1 in 2 prostheses and in zone 4 in 2 prostheses. In 1 prosthesis, a radiolucent line of 1–2 mm was present in zones 1 and 4, and in 1 prosthesis a radiolucent line of > 2 mm was present in zone 1 and one of 1 – 2 mm was present in zone 4. In another prosthesis, a radiolucent line of > 2 mm was present in both zone 1 and zone 4. Stress shielding was noted in zone 5 in 5 patients.

**Figure 3.**
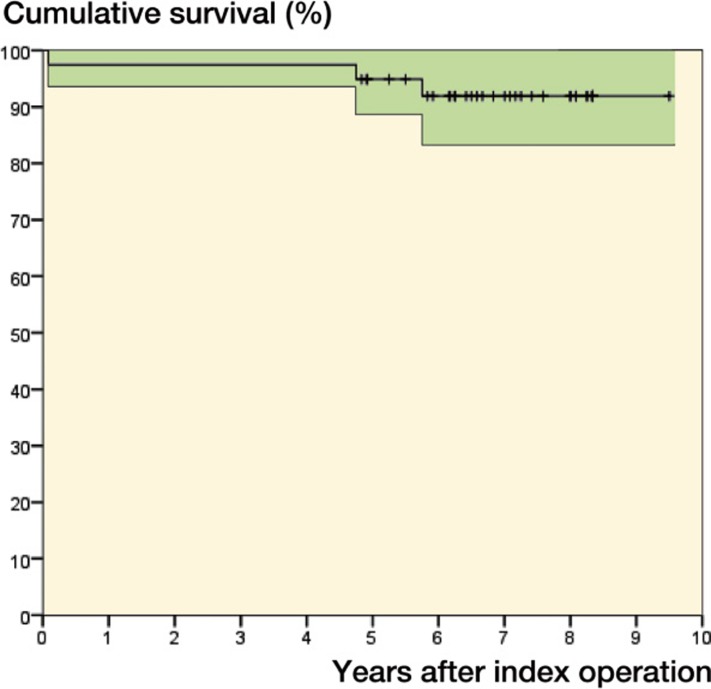
Kaplan-Meier survival curve (revision for any reason) of the CUT prosthesis (mean and 95% CI).

During follow-up, 3 stems were revised at 43 days, 4.8 years, and 5.8 years postoperatively for various reasons (Table 1). No stems were revised for aseptic loosening. The 5-year survival was 95% (95% CI: 87–100) (Figure 3).

**Table 1.** Details of revised CUT femoral neck prostheses

Revision	Primary diagnosis	Time to revision	Reason for revision	Remark
1: (F, 58 y)	Arthrogryphosis multiplex	43 days	Undersized femoral component	9.3 mm subsidence and subsequent luxation in a patient with a fork-deformed femur. A larger femoral component was implanted; this prosthesis stabilized and functions well.
2: (F, 35 y)	Septic monoarthritis	4.8 years	Persistent pain and bone resorption in zone 5 with suspected low-grade infection after 3.5 years	Stable prosthesis according to RSA analysis; intraoperative cultures negative for micro-organisms. After 6 weeks, a THA with diaphyseal stem was implanted.
3: (M, 51 y)	Osteoarthritis	5.8 years	Persistent postoperative pain	Stable prosthesis according to RSA analysis; no infection. Periarticulair ossification was present. Prosthesis was well-fixed at revision. A THA with diaphyseal stem was implanted.

### RSA results

**Figure 4.**
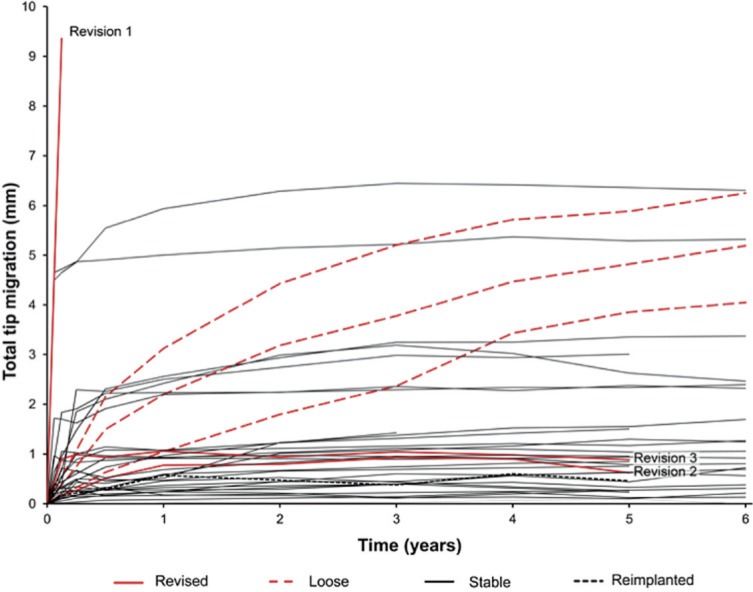
Total tip migration of 35 CUT prostheses. Represented are: 3 failures (solid red lines), 3 prostheses showing continuous excessive migration (dashed red lines), 29 stable unrevised prostheses (solid black lines), and 1 CUT prosthesis of larger size implanted in revision case 1 (dashed black line; see Table 1 for details).

**Figure 5.**
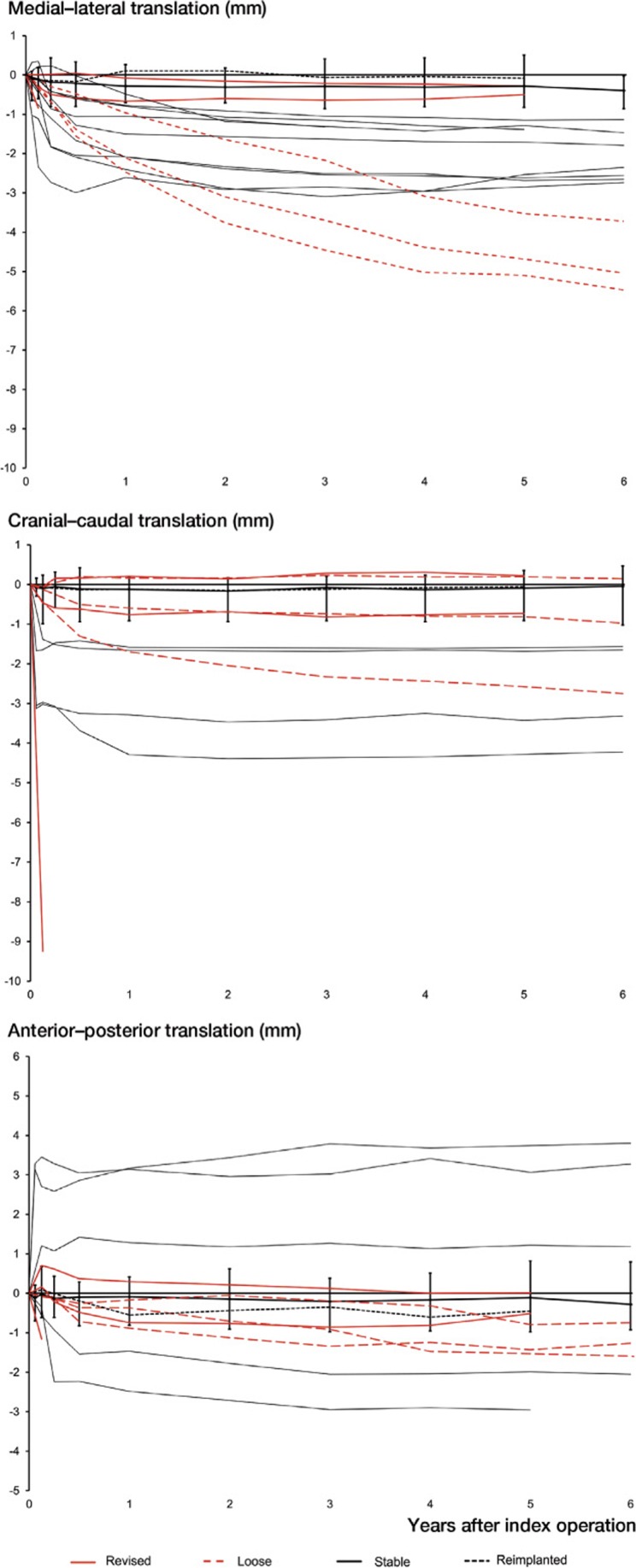
Medial-lateral (upper), cranial-caudal (middle), and anterior-posterior (lower) tip translation of the 35 CUT prostheses. For the 29 stable, unrevised prostheses, the mean translation with range (bars) is shown for those prostheses with translation below 1 mm; stable, unrevised prostheses with translation higher than 1 mm are shown as individual (solid black) lines. Also shown are 3 failures (solid red lines), 3 prostheses showing continuous excessive migration (dashed red lines), and 1 CUT prosthesis of larger size implanted in revision case 1 (dashed black line).

The initial migration varied widely in magnitude between different prostheses (Figure 4). The median total tip migration, i.e. the vector length, was 0.42 (0.09–9.36) mm at 6 weeks, 0.92 (0.18–5.94) mm at 1 year, and 0.89 (0.13–6.39) mm at 5 years. The overall initial migration pattern of the tip was composed of subsidence and lateralization (Table 2). Migration of more than 1 mm in the medial-lateral direction was measured for 13 prostheses (all 13 lateral), over 1 mm in the cranial-caudal direction for 8 prostheses (all 8 subsidence), and over 1 mm in the anterior-posterior direction for 8 prostheses (3 anterior, 5 posterior) (Figure 5).

**Table 2.** Migration of the CUT femoral neck prosthesis during the first 5 postoperative years. The medial, cranial, and anterior directions represent the positive directions

			Tip migration in mm (median and range)						
	6 weeks	3 months	6 months	1 year	2 year	3 year	4 year	5 year
Medial-lateral	–0.12 (–2.34 to 0.35)	–0.29 (–2.73 to 0.44)	–0.44 (–2.99 to 0.33)	–0.57 (–2.61 to 0.26)	–0.66 (–3.76 to 0.17)	–0.67 (–4.46 to 0.40)	–0.75 (–5.02 to 0.44)	–0.59 (–5.10 to 0.51)
Cranial-caudal	–0.09 (–9.26 to 0.24)	–0.10 (–3.10 to 0.31)	–0.09 (–3.68 to 0.42)	–0.10 (–4.29 to 0.21)	–0.13 (–4.39 to 0.17)	–0.08 (–4.37 to 0.28)	–0.10 (–4.34 to 0.31)	–0.09 (–4.22 to 0.35)
Anterior-posterior	–0.0 (–1.16 to 3.45)	–0.09 (–2.24 to 3.28)	–0.05 (–2.23 to 3.05)	–0.12 (–2.48 to 3.17)	–0.21 (–1.77 to 3.44)	–0.14 (–2.95 to 3.79)	–0.19 (–2.90 to 3.68)	–0.13 (–2.95 to 3.70)
Total translation	0.42 (0.09 to 9.36)	0.60 (0.11 to 4.87)	0.80 (0.07 to 5.54)	0.92 (0.18 to 5.94)	1.01 (0.17 to 6.29)	1.06 (0.11 to 6.45)	1.09 (0.14 to 6.42)	1.10 (0.13 to 6.39)

**Table 3.** Reported medium-term results of the CUT femoral neck prosthesis

Study	Number of THAs				Survival (%)		
	Mean length of follow-up (years)	Total number	Revised for any reason	Revised for aseptic loosening	At mean follow-up	At 5-year revision for any reason	At 5-year revision for aseptic loosening
Thomas et al. (2004)	3.5	136	4	4	97	–	–
Ender et al. (2007)	5.0	123	13	7	89	89	94
Rudert et al. (2007)	3.1	49	4	2	92	–	
Ishaque et al. (2009)	8.0	82	31	25	50	58	62–64
Steens et al. (2010)	6.6	99	2	1	98	98	98–99
Present study	7.0	39	3	0	92	95	100

Based on the total tip migration, 1 prosthesis showed rapid initial migration without any tendency to stabilize (Figure 4, revision 1). 3 additional prostheses in 3 patients showed continuous excessive migration, i.e. migration over the detection threshold of 0.25 mm on more than 1 occasion and continuing after 2 years of follow-up (Figure 4). These prostheses also showed the highest progressive migration rate in the medial-lateral direction (Figure 5). These 3 prostheses were considered to be loose. Furthermore, radiolucent zones in both zone 1 and zone 4 were present only in these 3 prostheses. This radiolucency in combination with the measured migration is consistent with progressive varisation of the prosthesis. When these 3 prostheses were considered to be additional failures, the 5-year survival for the combined endpoint revision and (radiographic) loosening was 86% (95% CI: 74–98).

It is noteworthy that these 3 prostheses already showed the highest total tip migration rate 6 months postoperatively, and that this remained the case throughout the entire 5 years of follow-up. Also, tip migration higher than 1 mm between 6 months and 2 years postoperatively was measured in these cases only: 2.28, 1.74, and 1.16 mm in these cases as opposed to migration below 0.89 mm for the stable prostheses (mean 0.27, range: 0.01–0.89).

Distance between the prosthesis and the proximal medial cortex and stem alignment were associated with the initial amount of medial-lateral migration of the tip (–0.76 mm per 2 mm distance, 95% CI: –1.26 to –0.26 (p = 0.005) and 0.06 mm per degree, 95% CI: 0.03–0.11 (p = 0.04)). Distance between the prosthesis and the lateral cortex was associated with initial longitudinal migration (0.52 mm per 2 mm distance, 95% CI: 0.02–1.02 (p = 0.04)). No factors were found to be associated with initial migration in the anterior-posterior direction. Also, no factors were found to be associated with the (continuous) steady-state migration in any of the 3 directions.

## Discussion

In this study, we analyzed the medium-term clinical and RSA results of 39 consecutive CUT femoral neck prostheses implanted for symptomatic osteoarthritis in young patients with heterogeneous and even rare underlying pathologies. 34 of 39 THAs had a good clinical outcome. 3 of the 39 prostheses were revised: 1 prosthesis was revised because of dislocation following excessive subsidence due to usage of an undersized component and 2 prostheses, which were both firmly fixed, were revised due to severe, persistent thigh pain. Severe persistent pain of the operated hip as a reason for revision is not uncommon after implantation of a CUT prosthesis, and has been reported by several authors (Ender et al. 2007, Ishaque et al. 2009, Steens et al. 2010). As such, it may be a characteristic of this particular prosthesis.

Using RSA, 3 additional prostheses were found to show continuous, excessive migration during the entire follow-up period and these prostheses were considered to be loose. Only these 3 prostheses showed radiolucent lines in zones 1 and 4, and the excessive continuous migration was mainly in the medial-lateral direction, which is compatible with the reported failure mode of the prosthesis—of increased horizontal migration and varisation (Ender et al. 2007, Ishaque et al. 2009, Steens et al. 2010). Unfortunately, no factors could be related to loosening: no excessive (medial) bone resorption was present and no gross abnormalities, regarding for example stem alignment or the amount of femoral neck resection, were noted. However, so far, revision has not been required for these 3 prostheses. The other 31 RSA-evaluable prostheses stabilized within the first preoperative year.

Several authors have reported medium-term survival of the CUT prosthesis (Table 3) (Thomas et al. 2004, Ender et al. 2007, Rudert et al. 2007, Ishaque et al. 2009, Steens et al. 2010). Our 5-year survival rate of 95% is in agreement with these reported rates. In these reports, aseptic loosening was the main reason for revision but its incidence varied considerably. This may be due to the different assessment times, variation in the decision about when to perform revision surgery, or difficulty in establishment of the diagnosis of aseptic loosing. Using RSA, the rate of aseptic loosening of implants can be established with a high degree of certainty. We identified 3 additional prostheses that were aseptically loose, and revision surgery may be necessary within the next 5 years. If so, the 10-year survival may be at best 83%.

Management of young patients requiring THA is difficult. Survival of conventional (uncemented diaphyseal) THA is generally lower than THA in the elderly, with 10-year survival of 88.5–91.0% in patients younger than 60 years reported by the Nordic arthroplasty registries (Eskelinen et al. 2005, Havelin et al. 2009). The results of resurfacing THA, often advocated for these patients, may be substantially inferior to those of diaphyseal THA (Duijsens et al. 2005, Springer et al. 2009, Johanson et al. 2010, Simpson and Villar 2010). A femoral neck prosthesis provides an alternative treatment option and has, like resurfacing THA, the potential to function as an intermediate step towards eventual placement of a diaphyseal THA. Minimal bone loss is required for implantation and, in the case of the CUT femoral neck prosthesis, the subsequent favorable strain distribution and prevention of proximal femoral stress shielding after implantation facilitate possible future revision THA. Medium-term survival of the CUT femoral neck prosthesis is satisfactory in these young patients and comparable to that of other femoral neck THAs (Zelle et al. 2004, Stukenborg 2007, Corner et al. 2008).

Our results do, however, suggest that the expected long-term survival of the CUT femoral neck prosthesis in these young patients may not reach the NICE-benchmark survival of 90% at 10 years (National Institute of Clinical Excellence (NICE) 2003). Furthermore, as noted by us and others (Ender et al. 2007, Ishaque et al. 2009, Steens et al. 2010), implantation of the CUT femoral neck prosthesis is a demanding procedure—even for skilled surgeons familiar with its implantation technique. Thus, at the moment, we cannot recommend the CUT femoral neck prosthesis as a routine treatment option in (young) patients requiring THA, and we should await the results of longer follow-up.

The present study demonstrates the feasibility of RSA-measured migration for assessment of performance of femoral neck prostheses at short-term follow-up. All 3 migrating prostheses that were found to be loose showed a distinctively high migration rate incompatible with stabilization between 6 months and 2 years of follow-up. Thus, these prostheses would also be indicated to have a high risk of future revision due to aseptic loosening at short-term follow-up, and analysis of the 2-year migration results would have led to similar conclusions regarding the expected numbers at risk of future revision due to aseptic loosening. This expected rate of aseptic loosening is certainly high enough to defer widespread introduction until the results of longer-term follow-up become available.

It is interesting to note that in case of the CUT femoral neck prosthesis, the magnitude of the initial migration cannot be considered to be the only predictive measure to assess fixation. This contrasts with common practice in RSA research, which is based on the findings for diaphyseal stems (Kärrholm et al. 1994, Hauptfleisch et al. 2006) and tibial components for total knee arthroplasty (Ryd et al. 1995). We found that large initial migration in either 1 of the 3 orthogonal directions could be compatible with subsequent stabilization of the prosthesis in that direction, but that a high migration rate during the first 2 years was indicative of failure of the implant to stabilize. This indicates that in terms of interpretation of RSA results with respect to possible future failure of an implant, it is not only the magnitude of the initial migration that should be assessed but also whether a plateau phase with stabilization of the implant has been reached.

It is recommended that the CUT prosthesis should have contact with both the medial and lateral femoral cortex (Rudert et al. 2007). Although a suboptimal position of the prosthesis was associated with increased initial migration, this did not influence subsequent stabilization of the prosthesis and a certain degree of suboptimal positioning appears to be tolerated. Unfortunately, we were unable to identify factors associated with continuous, excessive migration.

The fact that implant migration was measured using only 1 marker on the prosthesis tip was a limitation of our study: rotation of the prosthesis could not be measured. More specifically, although not necessarily disadvantageous, it is the movement of the prosthesis tip that is measured and not the movement of the (gravitational) center of the prosthesis. If the prosthesis migrates (rotates) *around* this tip marker, no migration is measured—although the prosthesis actually does migrate. However, this scenario is unlikely since the marker is located at the tip of the hook-shaped distal end, which is very unlikely to function as the (instantaneous) center of rotation of the prosthesis at any time. Another limitation of the study was the small study group and the absence of a formal power analysis, even though the sample was large enough for meaningful assessment of migration and survivorship.

In conclusion, at the moment we cannot recommend the CUT femoral neck prosthesis as a routine treatment option in (young) patients requiring THA. The CUT prosthesis may not reach the 90% survival benchmark at 10 years, and the prosthesis is difficult to implant. If initial stabilization is achieved, however, aseptic loosening is unlikely and a good clinical outcome is seen in the surviving prostheses. We will continue to follow this patient group.
